# Accelerated epigenetic aging in women with emotionally unstable personality disorder and a history of suicide attempts

**DOI:** 10.1038/s41398-023-02369-7

**Published:** 2023-02-22

**Authors:** Adrian Desai E. Boström, Peter Andersson, Esmail Jamshidi, Alexander Wilczek, Åsa Nilsonne, Mathias Rask-Andersen, Marie Åsberg, Jussi Jokinen

**Affiliations:** 1 Department of Clinical Sciences/Psychiatry, Umeå University, Umeå, Sweden; 2 Centre for Psychiatry Research, Department of Clinical Neuroscience, Karolinska Institutet, and Stockholm Health Care Services, Region Stockholm, Karolinska University Hospital, SE-171 76 Stockholm, Sweden; 3 Department of Clinical Neuroscience/Psychology, Karolinska Institute, Stockholm, Sweden; 4 Centre for Clinical Research Dalarna, Uppsala University, Falun, Sweden; 5 Department of Clinical Sciences, Karolinska Institutet at Danderyd Hospital, Stockholm, Sweden; 6 Department of Immunology, Genetics and Pathology, Science for Life Laboratory, Uppsala University, Uppsala, Sweden

**Keywords:** Physiology, Prognostic markers

## Abstract

Emotional unstable personality disorder (EUPD; previously borderline personality disorder, BPD) is associated with excess natural-cause mortality, comorbid medical conditions, poor health habits and stress related epigenomic alterations. Previous studies demonstrated that GrimAge – a state-of-the-art epigenetic age (EA) estimator – strongly predicts mortality risk and physiological dysregulation. Herein, we utilize the GrimAge algorithm to investigate whether women with EUPD and a history of recent suicide attempts exhibit EA acceleration (EAA) in comparison to healthy controls. Genome-wide methylation patterns were measured using the Illumina Infinum Methylation Epic BeadChip in whole blood from 97 EUPD patients and 32 healthy controls. The control group was significantly older (*p* < 0.0001) and reported lesser exposure to violent behavior in both youth and adulthood (*p* < 0.0001). Groups were otherwise comparable regarding gender, BMI, or tobacco usage (*p* > 0.05). EA estimator DNAmGrimAge exceeded chronological age by 8.8 and 2.3 years in the EUPD and control group, respectively. Similarly, EAA marker AgeAccelGrim was substantially higher in EUPD subjects when compared to controls, in both univariate and multivariate analyzes (*p* < 0.00001). Tobacco usage conferred substantial within-group effects on the EA-chronological age difference, i.e., 10.74 years (SD = 4.19) compared to 6.00 years (SD = 3.10) in the non-user EUPD group (*p* < 0.00001). Notably, past alcohol and substance abuse, use of psychotropic medications, global assessment of functioning, self-reported exposure to violent behavior in youth and adulthood, later completed suicide (*N* = 8) and age at first suicide attempt did not predict EAA in the EUPD group (*p* > 0.05). These results underscore the importance of addressing medical health conditions along with low-cost preventative interventions aimed at improving somatic health outcomes in EUPD, such as efforts to support cessation of tobacco use. The independency of GrimAge to other EA algorithms in this group of severely impaired EUPD patients, suggest it may have unique characteristics to evaluate risk of adverse health outcomes in context of psychiatric disorders.

## Introduction

Emotionally unstable personality disorder (EUPD), also referred to as Borderline Personality Disorder, is a mental disorder marked by a persistent pattern of unstable and intensive interpersonal relationships, efforts to avoid real or perceived abandonment, affective instability, identity disturbance and self-destructive impulsive behavior [1]. EUPD is a highly impairing condition, entailing significant disease burden on those afflicted [2]. Longitudinal studies report that psychiatric comorbidity is more widespread in EUPD than in other personality disorders [3, 4], with observed prevalence rates of 90% for any other co-occurring psychiatric disorder [5]. As in other mental illnesses, mortality is elevated in EUPD, exemplified by reports of a nearly five-fold increase in all-cause mortality in comparison to the general population (standardized mortality ratio = 4.8) [6]. Indeed, premature death has been reported to be more common in EUPD than in other personality disorders [7]. One obvious cause of elevated mortality levels is the suicidal behavior that is intrinsic to the condition: with some studies reporting up to 50-fold increases in suicide risk in EUPD-populations [8]. However, even accounting for this and death by other unnatural causes such as accidents, substantial excess mortality has been observed [9]. Additionally, EUPD is associated with a wide variety of medical conditions, such as hypertension and other cardiovascular diseases, sexually transmitted diseases, arthritis, obesity, physical pain and sleep disturbances [10–12]. Previous studies also demonstrate that EUPD is associated with a high prevalence of poor health habits, i.e., smoking, physical inactivity, and overconsumption of medicine [13]. Additionally, substance abuse disorder has been observed to co-occur in over 50% of EUPD cases [14]. Although non-psychiatric health care utilization is high in EUPD [15], a decreased compliance to treatment regimens has been suggested as a putative cause of poor health outcomes [16].

Dysregulation in several neurobiological systems has been suggested in pathophysiological models of EUPD [17]. Examples of proposed biological mechanisms at play include the endogenous opioid theory of EUPD, which describes symptoms such as dysphoria, sensitivity to social rejection, deficient emotional regulation and non-suicidal self-injury as underpinned by dysfunction in the endogenous opioid and the dopaminergic systems [18]. The interpersonal difficulties and maladaptive stress responses characteristic of the disorder have also been suggested to be caused by abnormalities in the oxytocinergic system [19]. Disequilibrium in the stress regulatory hypothalamic-pituitary-adrenal axis (HPA-axis) has been extensively studied in the context of EUPD [20]. Previous studies implicate both functional [21] and epigenetic alterations [22] in this important neuroendocrine system, suggestive of biological mechanisms underlying emotional instability in EUPD.

The calculation of epigenetic aging (EA) is an emerging approach to study the impact of intrinsic and extrinsic factors on health, facilitated by advances in epigenetic research focused on DNA methylation, a process central to epigenetic regulation of gene expression [23]. EA is more closely related to overall health status and cellular senescence than chronological age and permit estimation of accelerated biological aging, i.e., when estimated biological age exceeds chronological age, accelerated biological aging, indicative of poor health status, can be deduced. Previous work in populations with severe mental disorders have attempted to gauge the health effects of mental illnesses by measuring EA. Thus far, deducing robust causal inferences from these efforts is complicated by underpowered studies, heterogeneous measurement methodologies employed and insufficient rigor in the deep phenotyping of included subjects [24]. Several “epigenetic clocks” designed to measure EA, have been proposed (i.e., for example, Horvath’s Clock [25], Hannum Age [26], DNAmPhenoAge [27]). Whilst many of these measures predict chronological age, morbidity and excess mortality [23], correlations amongst EA measures are weak [28]. This has been interpreted as indicative of several different processes underpinning biological aging, with different EA estimators putatively reflecting different aspects of the multifaceted aging process [29]. To address the so-called “epigenetic clock” conundrum, Lu. et al. recently presented the “DNAm GrimAge” EA measure. The GrimAge algorithm is based on consolidated surrogate DNA methylation levels used to estimate plasma protein levels and has been demonstrated to be a strong predictor of lifespan [28]. In direct comparisons with other EA algorithms, GrimAge was superior to all other methods in prediction of time-to-death, time-to-cancer (any) and time-to-coronary disease in three validation datasets comprising >7000 array measurements. In addition, this EA/A estimate is strongly correlated to radiology-based estimates of excess visceral fat and a comorbidity index (defined as the total number of age-related conditions). AgeAccelGrim – a measurement of epigenetic age acceleration (EAA) obtained by adjustment of GrimAge to chronological age, strongly predicts onset of age-associated medical conditions such as time-to-congestive heart failure, hypertension, type 2 diabetes mellitus, physical functioning and time-to-congenital-heart-defects (CHD) [28].

The field of epigenetic aging and its connection to psychiatric populations is a growing area of research. However, the impact of EUPD on the state-of-the-art epigenetic age acceleration algorithm, GrimAge, has yet to be studied in relation to patients with a history of recent suicide attempts. Our aim was to investigate this relationship by comparing a cohort of EUPD patients to a group of psychiatrically healthy controls. We included smoking as a predictor, as it is a well-known epigenetic risk factor that can affect DNA methylation patterns and contribute to cellular aging. The study included multiple epigenetic age clocks, including GrimAge, to provide a comprehensive evaluation of epigenetic aging in the study population. In addition, the impact of key clinical variables on epigenetic age acceleration was examined in order to identify additional risk factors for premature mortality in this severely affected EUPD patient cohort and comparison group of psychiatrically healthy volunteers.

## Methods

### Study population and ethics

Details on the EUPD cohort have been previously published [30–32]. The original study protocol was approved by the Committee for Ethical Research at Karolinska Institutet (Dnrs: 95–283; 2021-06929-01). In brief, the patient group consisted of 162 unrelated Caucasian females living in the Stockholm County, originally recruited as part of a randomized controlled trial (RCT) comparing two psychotherapy modalities (dialectical behavioral therapy and psychodynamic therapy) to treatment as usual (TAU) in patients with a history of suicidal behavior. Patients were referred from all Stockholm County Council psychiatric clinics (encompassing care of 1.8 million inhabitants) for a dialectic behavioral therapy (DBT) project called SKIP. A total of 162 women with BPD were invited to take part in the SKIP project. Of these individuals, 14 declined to join the study, 41 were excluded due to not fulfilling inclusion criteria or to fulfilling exclusion criteria and one completed suicide before joining the study. Thus, out of 162 women, 106 (65%) took part in the SKIP study and blood samples were available for 97 patients. Blood samples were collected before the interventional point of the study, i.e., prior to randomization to any treatment modality.

Study inclusion criteria included a prior history of two or more potentially lethal suicide attempts (as defined by the patient’s belief that the attempt could have been lethal), with at least one attempt during the six months preceding referral. Subjects were excluded if presenting with a current life-threatening eating disorder, current psychotic disorder or major depressive illness with melancholic features, evidence of dementia or other irreversible organic brain syndrome or an active diagnosis of substance dependence [32]. The Structured Clinical Interview for DSM-IV Axis I and II interview (SCID-I and II) schedules and the Comprehensive Psychopathological Rating Scale interview were administered at baseline and EUPD diagnosis and psychiatric comorbidities were established after consensus diagnostic conference amongst experienced clinicians. Subjects were required to be in the 18–50-year-old age group. The baseline measurements of this trial included, amongst other things, a blood sample, which was analyzed to extract epigenetic data in the current project. Data from various clinician rated diagnostics interviews, self-report assessments and cognitive tests were collected at baseline. Through the unique personal identification number in Sweden, patients were linked to the Cause of Death Register maintained by the Swedish National Board of Health and Welfare. Eight suicides were ascertained from the death certificates. Blood samples were collected between 1999 and 2004 and follow-up times ended 2011.

The control cohort was originally recruited from a set of healthy females in a separate project [33], approved by the Regional Research Ethics Review board of Stockholm (Dnrs:2008/4:2). Participant exclusion criteria for this group included any psychiatric morbidity or serious medical illness such as stroke, cancer, or a previous myocardial infarction. Measurements included a blood sample at baseline, which was analyzed to extract epigenetic data in the current study. Absence of psychiatric symptoms was ascertained by administration of the SCID-I by an experienced clinician. Blood samples for the control cohort were collected between 2009 and 2010.

Participants in both groups gave written informed consent.

### Blood sample collection, DNA methylation profiling and preprocessing

Standard procedures were utilized in blood sampling. Extraction from non-fasting subjects occurred in the morning. Retrieval of DNA from 97 EUPD participants and 32 control subjects was conducted by the phenol-chloroform method and samples were subjected to bisulfite conversion according to the EZ DNA Methylation GoldTM kit (ZymoResearch, USA). Hybridization of the resulting DNA to the Illumina Infinium Methylation EPIC BeadChip was executed. Thereafter, methylation values for 850 K probes for each sample was obtained through array imaging by Illumina iScan system (Illumina, San Diego, CA, USA). Specialized instructions for EA estimation following guidelines from recent publications [34] were implemented in the preprocessing of methylation data. Quality control and normalization of raw methylation IDAT data was conducted with the mefill package for R statistics (https://github.com/perishky/meffil/), utilizing control probes to isolate biological variation from technical. All samples passed QC, resulting in extraction of methylation β-values in a total of 129 subjects (97 EUPD and 32 control subjects).

### DNAm age calculation

Data from 27,253 CpG sites was uploaded to the DNAm Age Calculator (https://dnamage.genetics.ucla.edu/). 2562 probes from the list of 30,085 probes recommended by Horvath et al. were unaccounted for. The non-complete CpG-site overlap between the EPIC and 450k Illumina platform was the reason for this discrepancy, with the present study utilizing the EPIC Beadchip (epigenetic clocks are derived from the 450k BeadChip) [34]. A complementary dataset containing data on chronological age, gender, and sampling tissue, was uploaded to perform the “Advanced Analysis”.

Several EA measures – representing unique epigenetic clock algorithms – were extracted from the 129 study subjects. These measures included epigenetic age acceleration by Horvath and Hannum (IEAA [35] and IEAAHannum [26]) and extrinsic epigenetic age acceleration (EEAA [35], AgeAccelPheno [27] and AgeAccelGrim [28] - the two latter derived from independent estimators [28, 36]). EA acceleration measures are directly interpreted, i.e., a value greater than 0 implicates EA exceeding chronological age, and vice versa. Extraction of measures of intrinsic age acceleration (IEAA) was also performed. In contrast to EAA, these represent estimates that are independent from alterations in blood cell type composition and are associated with the aging process [33]. Age-adjusted DNAmTL (DNAmTLadjAge), is a measure based on telomere length that is interpreted inversely, i.e., a negative DNAmTLadjAge suggest shorter than expected DNAm-based estimates of telomere length, indicating accelerated EA. Different epigenetic age predictors have been extensively described previously [34].

### Statistical analysis

To detect between-group differences in demographic and clinical variables, independent samples *t*-test, chi-squared test and Fisher’s exact test were utilized. Analysis of normality in investigated measures of EA acceleration was performed by Shapiro-Wilk test, which indicated normal distributions in all measures except for DNAGrimAge and AgeAccelGrim. These two measures were thus subjected to Blom-transformation [35], to achieve normality for uni- and multivariate analyses (not used for visualization). Interrelatedness of epigenetic clocks and their association to chronological age was investigated by Pearson correlations across both the full study sample and sub-samples (i.e. EUPD and control subjects, respectively) [29, 37], in order to assess the accuracy of derived EA/A estimators. Mean difference between DNAmGrimAge and chronological age was assessed for each group separately, with percentage change in relation to chronological age visualized in violin plots for illustrative purposes. To prevent bias from overfitting by inclusion of too many-covariates, only parameters associated with AgeAccelGrim in the EUPD-subgroup with a *p*-value < 0.10 were included (such analyses excluded pre-specified group differences, i.e., frequency of psychiatric diagnoses, psychotropic medications, history of suicide attempt etc.). Associations between demographic and clinical variables and AgeAccelGrim in the EUPD-group were thus evaluated by Pearson correlations for continuous variables, Spearman’s rank correlation coefficient for ordinal variables, and independent samples *t*-test in the case of dichotomous variables. Robust linear regression models using the *robustbase* package for R statistics [36] were implemented where linear regressions could be confounded by outliers. Active or previous tobacco use was significantly associated to AgeAccelGrim (*p* = 6.4E-08). Chronological age was not significantly associated to AgeAccelGrim at a designated standard *p*-value threshold of 0.05 but was included as a co-variate due to the more lenient association threshold chosen for determining optimal co-variates (i.e., *p* = 0.09). Other demographic or clinical characteristics did not correlate significantly with AgeAccelGrim (*p* > 0.1). Thus, clinical categorization (EUPD or control), active or previous tobacco use and chronological age, were selected as co-variates for the multivariate model. Significant between-group differences were discovered in the case of several clinical characteristics, but these were not considered for inclusion as co-variates due to perfect co-linearity with the grouping variable (EUPD or control, see above). To assess power to detect meaningful differences in AgeAccelGrim in relation to clinical categorization, the ‘power-*t*-test’ function for R statistics was implemented. Two-tailed hypothesis *t*-tests were utilized in investigation of between group differences in EA measures by suicidal risk group. Multiple linear regression models were used in estimations of associations of AgeAccelGrim, the clinical grouping variable and optimal co-variates, contrasting GrimAgeAcceleration to clinical group (EUPD or control), active or previous tobacco use and chronological age. To further investigate any associations between global assessment of functioning (GAF) and EAA, we performed independent samples *t*-tests contrasting GrimAge-derived EAA-measures to a dichotomized GAF-variable (Cut-off: <36).

## Results

### Baseline descriptives

The 97 EUPD participants were all females with a mean age and BMI of 29.4 years (SD = 7.6) 24.5 kg/m^2^ (SD = 4.7), respectively. Approximately a third had attained a university-level education and 10.3% had biological children. Active or previous tobacco usage was recorded in 57.7% of the EUPD-group. All subjects fulfilled criteria for one or more DSM-IV Axis I psychiatric diagnoses. Anxiety disorders were most commonly prevalent (60.8%) and a substantial proportion had active major depressive disorder (MDD, 42.3%), of which a subset presented with severe MDD (13.4%). Naturally, all participants met criteria for EUPD, 32% of which fulfilling criteria for 7 or more items. Subjects had a mean global assessment of functioning (GAF) score of 49.2, corresponding to serious symptoms or serious impairment in social, occupational, or school functioning [38]. History of alcohol and substance abuse were highly prevalent (33.0% and 26.8%, respectively). Bensodiazepines constituted the most frequent psychotropic medication (36.1%), closely followed by selective-serotonin reuptake inhibitors (SSRI; 33.0%), non-SSRI antidepressants (20.6%), neuroleptics (12.4%) and mood stabilizers (4.1%). All subjects had a history of recent suicide attempts (constituting inclusion criteria), whereby the mean age at first suicide attempt was 20 years (SD = 7.5). Eight women (8.25%) died by suicide after their participation in the study was concluded, in the years 2002–2012. These deaths occurred by intoxication (*N* = 5), hanging (*N* = 2) and railway suicide (*N* = 1). One additional subject died of unknown cause, which was not categorized as a suicide-related death.

The control group consisted of 32 female participants with a mean age and BMI of 37.2 years (SD = 6.0) and 24.1 kg/m^2^ (SD = 3.7), respectively. A majority had biological children and 40.6% exhibited ongoing or previous tobacco usage. Data on educational attainment and GAF was not available. Subjects were overall psychiatrically and physically healthy, presenting with no Axis I or II psychiatric diagnosis – and using no psychotropic medication. No control subject had previously attempted suicide. There were no significant differences between the groups regarding gender, BMI, or tobacco usage (*p* > 0.05). However, the control group were of significantly greater age, more often had biological children and reported on lower exposure to violent behavior in both youth and adulthood (*p* < 0.0001). There were also substantial (pre-specified) differences in frequencies of psychiatric diagnoses, use of psychotropic medications and history of past suicidal events (Table 1).

**Table 1:** Characteristics of subjects.

	BPD	Control	Statistics (*t*-test, Chisq-test, Fisher’s exact test), *p*-value
N	97	32	
Age (years), mean (SD)	29.4 (7.6)	37.2 (6.0)	<0.0001
Men:women, *n* (%)	0 (0.0): 97 (100.0)	0 (0.0): 32 (100.0)	ns
Biological children, *n* (%)	10 (10.3)	21 (65.6)	<0.0001
University Education, *n* (%)	28 (28.9)	N/A	-
BMI, mean (SD)	24.5 (4.7)	24.1 (3.7)	ns
Tobacco usage, *n* (%)	56 (57.7)	13 (40.6)	0.075
Active Major Depressive Disorder, *n* (%)	41 (42.3)	0 (0.0)	-
Severe MDD, *n* (%)	13 (13.4)	0 (0.0)	-
Bipolar Disorder II or UNS, *n* (%)	8 (8.2)	0 (0.0)	-
Comorbid Anxiety Disorder, *n* (%)	59 (60.8)	0 (0.0)	-
Borderline personality disorder, *n* (%)	97 (100.0)	0 (0.0)	-
-≥7 fullfilled BPD criteria, *n* (%)	31 (32.0)	0 (0.0)	-
History of alcohol abuse, *n* (%)	32 (33.0)	0 (0.0)	-
History of substance abuse, *n* (%)	26 (26.8)	0 (0.0)	-
KIVS exposure to violent behavior during childhood (6–14 years of age), mean (SD)	2.66 (1.86)	0.36 (0.71)	<0.0001
KIVS exposure to violent behavior as adult (>15 years of age), mean (SD)	2.47 (1.90)	0.35 (0.76)	<0.0001
Global Assessment of Functioning (GAF), mean (SD)	49.2 (12.4)	N/A	**-**
Psychotropic Medication, *n* (%)			
SSRI	32 (33.0)	0 (0.0)	-
Non-SSRI antidepressants	20 (20.6)	0 (0.0)	-
Mood stabilizers	4 (4.1)	0 (0.0)	-
Bensodiazepines	35 (36.1)	0 (0.0)	-
Neuroleptics	12 (12.4)	0 (0.0)	-
History of past suicide attempt, *n* (%)	97 (100.0)	0 (0.0)	-
Age at first suicide attempt, mean (SD)	20.0 (7.5)	-	-
Later confirmed death by suicide, *n* (%)	8 (8.25)	0 (0.0)	0.20

*P*-values were calculated by means of *t*-test, Mann-Whitney *U*-test or chi-squares test, contrasting values for control and BPD subjects. Pre-specified group differences were not subjected to such analyzes, i.e., frequency of psychiatric diagnoses, psychotropic medications or history/age at first suicide attempt. Subjects with extensive past or ongoing tobacco usage were classified as users, whereas occasional/party smokers (and non-users) were categorized as non-users. History of alcohol or substance abuse was considered present when the diagnosis of an alcohol or substance abuse diagnosis was recorded (incuding cases where these were in remittance). Non-SSRI antidepressant in the majority pertained to ongoing usage of psychotropic drugs with active substances desvenlafaxine (Effexor), desmethylmirtazapine (Remeron/Mirtazapine) and Amitriptyline (Clomipramine)(in order of frequency). Mood stabilizers included Valproic Acid (Ergenyl), Litium (Lithionit) and Lamictal (Lamotrigine). Bensodiazepine medications used were alprazolam (Xanor), clomethiazole (Heminevrin), Oxazepam (Sobril), Diazepam (Stesolid) - also Z-drugs Zopiclone (Imovane) and Zolpidem (Stilnoct) were included. Neuroleptics included Levomepromazine (Nozinan), Risperidone (Risperdal), Olanzapine (Zyprexa), Haloperidol (Haldol) and Quetiapine (Seroquel). The number of recorded suicidal/parasuicidal events did not take severity of attempt into account (i.e., intoxications or self-cutting were weighted equally to more severe attempts involving, for example, violence). A one-tailed *p*-value < 0.05 was considered significant.

*BMI* body-mass index, *BPD* borderline personality disorder, *KIVS* karolinska interpersonal violence scale, *ns* not significant, *SSRI* selective serotonin reuptake inhibitor.

### Interrelatedness between epigenetic clocks and their association to chronological age

**Fig. 1:**
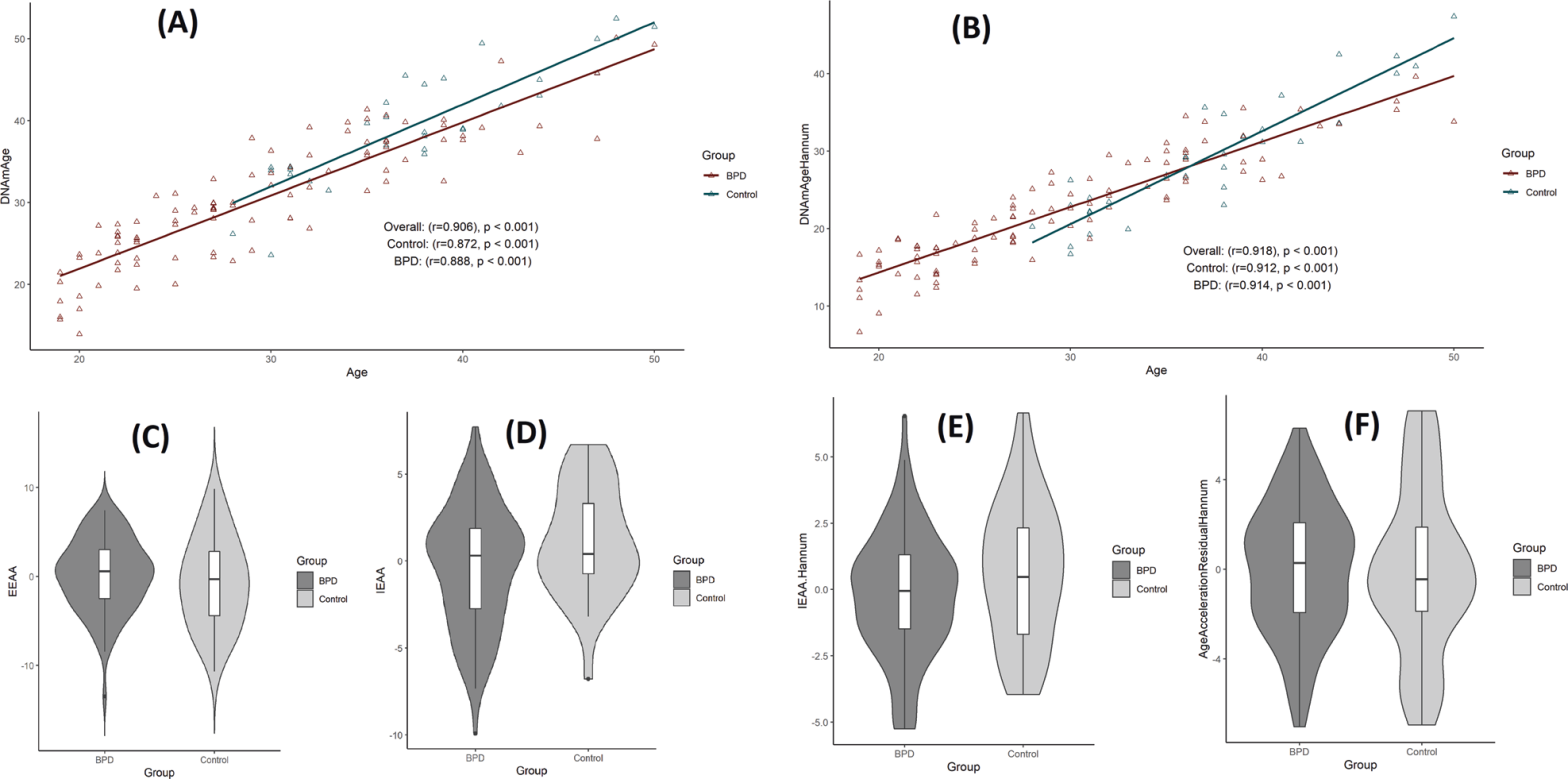
Horvath and Hannum epigenetic age acceleration. **A**, **B** Scatterplots show Horvath or Hannum Age vs chronological age. Pearson’s correlation analysis indicated a significant correlation between DNA methylation age and chronological age in both groups. **C**–**F** Violin plot with boxplots show Horvath EAA, IEAA, Hannum EAA, or EEAA. Between-group comparisons were conducted using a Student’s *t*-test. No significant between group differences were revealed (*p* > 0.1). EAA epigenetic age acceleration, IEAA intrinsic epigenetic age acceleration, EEAA extrinsic epigenetic age acceleration.

**Fig. 2:**
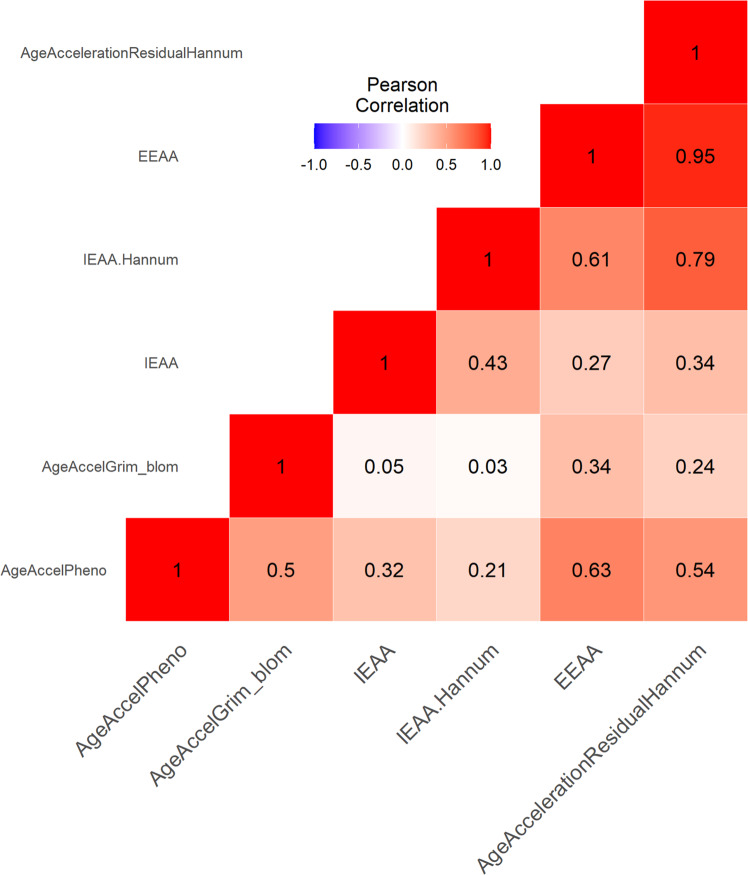
Correlations of AgeAccelGrim with other epigenetic age clocks. The plot visualizes the inter-correlations between seven DNAm clocks. The deeper color indicates stronger correlations. AgeAccelGrim was relatively independent to other clocks, while AgeAccelPheno was correlated of medium strength with the acceleration of Horvath and Hannum clocks.

**Fig. 3:**
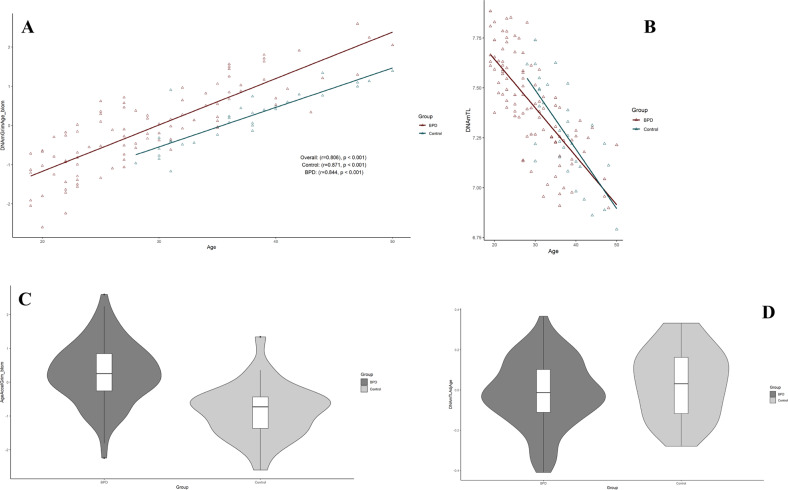
Grim epigenetic age acceleration and DNA methylation-based telomere length. **A**, **B** Scatterplots show GrimAge or DNAmTL vs. chronological age. Pearson’s correlation analysis indicated a significant correlation between GrimAge/DNAmTL and chronological age in both groups. **C**, **D** Violin plot with boxplots shows Grim EAA or DNAmTLAdjAge. Student’s *t*-test demonstrated significantly higher GrimAgeAccel in EUPD subjects (*p* =< 0.00001). EAA epigenetic age acceleration, DNAmTL DNA methylation-based telomere length, DNAmTL AdjAge age-adjusted DNAmTL.

Analyses revealed significant and strong correlations between chronological age and Horvath Age (*r* = 0.906, *p* < 0.001), Hannum Age (*r* = 0.918, *p* < 0.001), GrimAge (*r* = 0.806, *p* < 0.001), and DNAmTL (*r* = −0.753, *p* < 0.001) (Figs. 1, 3). A valid high accuracy of the epigenetic estimators used in the study is indicated by strong linear relationships between each DNAm age/DNAmTL measure and chronological age. A moderate but significant correlation between AgeAccelGrim and AgeAccelPheno (*r* = 0.50, *p* < 0.0001) was observed, in line with previous findings [28, 29]. AgeAccelGrim was relatively independent to other EA estimators, whilst a moderate-strength correlation was evinced between AgeAccelPheno and acceleration of the Horvath and Hannum clocks. The Horvath and Hannum clocks exhibited strong correlations amongst themselves (*r* > 0.5, *p* < 0.001), including both intrinsic and extrinsic measures of EAA (Fig. 2).

### Relatedness of AgeAccelGrim and other epigenetic age acceleration clocks with EUPD

Mean differences between DNAmGrimAge, and chronological age was 7.2 years (SD = 5.02 years) in the full cohort. EA disparities were substantially larger in the EUPD-group (mean difference = 8.8 years, SD = 4.48), in comparison to controls (mean difference = 2.3 years, SD = 3.0 years). Percentage change in DNAGrimAge compared to chronological age averaged 6.6% in controls and 32.2% in the EUPD-group (Supplementary Fig. 1). The power-analysis support the study’s ability to adequately detect differences of 2.3 years in AgeAccelGrim (desired power level specified 0.8) between the two groups for one-tailed hypothesis *t*-tests (Supplementary Fig. 2) and 2.5 for two-tailed hypothesis *t*-tests (data not shown). In the primary analysis, association of various estimators of EAA was assessed in relation to group stratification (EUPD/control) by univariate analyses. These analyzes demonstrated that EAA was independent of EUPD for most EA measures (i.e., EAA, IEAA, Hannum IEAA, AgeAccelerationResidualHannum and DNAmTLadjAge) (*p* > 0.1). The only epigenetic clock algorithm evincing an association between EUPD and EAA was AgeAccelGrim, which demonstrated substantial effects (*p* < 0.00001) (Figs. 1, 3). EUPD and tobacco usage were the strongest predictors of Grim Age Acceleration (β = 1.13 and 0.83, respectively; *p* < 0.000001), as measured by multiple linear regression models contrasting AgeAccelerationGrim to group (EUPD or control) and optimal co-variates. In this analysis, chronological age exhibited significant but substantially smaller effects (β = 0.02; *p* = 0.0205) (Table 2). Despite the strict Bonferroni correction method – adjusting for the iterations of epigenetic age that were evaluated – the results of our study still showed a significant association between EUPD and tobacco usage with EAA as measured by AgeAccelGrim (*p* < 0.00001).

**Table 2:** Multiple linear regression model contrasting Grim Age Acceleration to group (EUPD or control) and optimal co-variates.

	Estimate	Std. Error	*t*-value	*p*
Intercept	−1.98	0.36	−5.49	**2.30E-07**
Group (EUPD or Control)	1.13	0.17	6.69	**7.43E-10**
Tobacco usage (Y/N)	0.83	0.14	6.14	**1.11E-08**
Chronological age (years)	0.02	0.01	2.35	**2.05E-02**

Multiple linear regression model contrasting Grim Age Acceleration to group (EUPD or control) and adjusting for optimal co-variates (history of regular or active tobacco usage [dichotomous] and chronological age [continuous]. Shown are the coefficients and *p*-values. Significant findings are highlighted in bold (*p* < 0.05).

Abbreviations: *p* *p*-value.

### Relatedness of AgeAccelGrim with clinical variables in the EUPD group

To determine within-group factors of influence on epigenetic age acceleration, we tested all clinical variables presented in Table 1. for an association with AgeAccelGrim by Pearson correlations or independent samples *t*-tests (in cases of continuous and dichotomous variables, respectively). Robust linear regression models (RLRM) were utilized in instances where outliers could confound results of linear regressions [39]. Significant associations between active or previous tobacco usage and AgeAccelGrim were revealed (*p* = 6.4E-08), with means of −0.60 (SD = 0.68) and 0.42 (SD = 0.93) in the control and EUPD group, respectively. No other significant correlations between demographic or clinical characteristics and AgeAccelGrim were observed. Notably, KIVS-derived estimates of exposure to violent behavior in both youth and adulthood were weakly but significantly correlated to AgeAccelGrim across the full study sample (Spearman’s rho: 0.27, *p* < 0.005) – findings that were not replicated in sub-analyzes pertaining exclusively to the EUPD or control group (*p* > 0.05) (data not shown). As a post-hoc analysis, we sought to investigate previous findings implicating increased GrimAge-derived EAA estimates in relation to GAF [40]. Thus, in the EUPD-group, AgeAccelGrim was contrasted in a one-sided Mann-Whitney *U*-test to a dichotomized GAF-variable (cut-off: <36) in 11 individuals with mean GAF 28.9 (SD = 7.55) and 118 individuals with mean GAF 52.87 (SD = 9.11) – resulting in significant between-group differences (W = 212, *p* = 0.0271) (data not shown).

## Discussion

We present the first study implementing state-of-the-art EAA algorithms in a well-characterized cohort of severely suicidal EUPD patients. This study adds to previous findings implicating excess natural-cause mortality, high prevalence of comorbid medical conditions, poor health habits and stress related genomic alterations in EUPD. Specifically, in this cohort of women with EUPD and psychiatrically healthy control subjects primarily in early adulthood, DNAmGrimAge exceeded chronological age by 8.8 and 2.3 years in the EUPD and control group, respectively – conferring a mean EUPD-associated cumulative increase in biological aging of 32.2% compared to chronological age. Notably, other EA algorithms were not successful in evincing any between-group differences in EA/EAA. Surprisingly, several factors that could be presumed to confer large effects on excess morbidity and mortality – i.e., previous alcohol and/or substance abuse, use of psychotropic medications, age at first suicide attempt or self-reports on exposure to violent behavior in youth or adulthood - did not predict EAA in the EUPD group. Notably, tobacco usage conferred substantial within-group effects on the EA-chronological age difference, i.e., 10.74 years (SD = 4.19) compared to 6.00 years (SD = 3.10) in the non-user EUPD group (*p* < 0.00001). These results underscore the importance of addressing somatic health conditions in EUPD patients. The large effect of tobacco usage on EAA measures implicate targeted smoking cessation efforts across the lifespan have potential as low-cost interventions to reduce excess somatic morbidity and non-suicidal mortality. The independency of GrimAge to other EA algorithms in this group of severely impaired EUPD patients, suggest it has unique characteristics to evaluate risk of adverse health outcomes in context of psychiatric disorders.

The increased attention to medical risk factors and comorbid somatic conditions in psychiatric populations has been previously emphasized to reduce excess mortality and morbidity [41]. The present study further highlights the importance of these endeavors in the context of EUPD. The results showed that the somatic health effects associated with EUPD are severe, as demonstrated by the 32.2% increase in EA measured by the state-of-the-art algorithm GrimAge, compared to chronological age in a sample of female EUPD patients in early adulthood. This highlights the need for both clinical attention and further research in this area. To develop effective interventions to prevent and alleviate negative health effects in EUPD, it is crucial to have a coherent model of the mechanisms underlying the association between EUPD psychopathology and somatic morbidity. This study showed that many clinical variables, previously believed to contribute to somatic comorbidities and excess non-suicidal mortality in psychiatric populations, were not related to EAA (Epigenetic Age Acceleration) in the group of EUPD patients, except for current or past frequent tobacco usage. For example, past abuse of illicit substances and/or alcohol was not associated with EAA in this group of EUPD patients, which contrasts with previous studies [42, 43]. This discrepancy could be due to the high number of risk factors observed in this group of severely impaired EUPD patients with a recent suicide attempt. The study confirmed that there was no association between psychotropic medication usage and epigenetic age, which is consistent with prior research that found no evidence of a connection between psychotropic medication use and DNA methylation at the loci used to estimate AgeAccelGrim [44]. The results also indicated that GAF (Global Assessment of Functioning) scores did not follow an ordinal-linear relationship to EAA in this cohort of EUPD patients. However, preliminary evidence suggested that the most severely impaired subjects showed increased GrimAge acceleration in relation to GAF. The study results indicate that self-reported exposure to violence during both youth and adulthood did not have a significant effect on EAA estimates derived from GrimAge, challenging previous findings that linked childhood adversity to inferior EAA measures [45, 46]. It should be noted that these previous studies utilized alternative EAA estimation algorithms that have weaker associations with mortality indexes compared to GrimAge [28]. The age at first suicide attempt was also independent of GrimAge-derived estimates of EA/A, which was surprising given that it has been previously implicated as impacting related reductions in life expectancy [45]. Overall, the results of the study emphasize the need to develop effective interventions to prevent and alleviate negative health effects in EUPD.

One putatively contributing factor to increased EAA in EUPD are adverse environmental factors, such as poor health habits that are common to the disorder. Previous studies have indicated associations between EAA and smoking [46] and low levels of non-occupational physical activity [47] – adverse life-style habits previously shown to be highly prevalent in EUPD [10]. Other extrinsic factors (not investigated in this study) that could be of interest in the future exploration of the relationship between EUPD and EAA, are sleep disturbances. Preliminary evidence from a small cohort of female college students found prospective effects on EAA in poor sleepers [48] and obstructive sleep apnea was recently evinced as a reversible cause of EAA [49]. This may be of some relevance, especially so as objective and subjective sleep disturbances in EUPD are comparable in scope and scale to those found in major depressive disorder [50]. Another possible contributing factor underlying the association between EUPD and EAA are the putative neurobiological alterations presumed to be more endogenous to the condition. For example, alterations in HPA-axis functioning have been theorized to play an important role in the pathophysiology of EUPD and have been extensively studied, evincing both lower basal cortisol levels [20] and altered cortisol responses to psychosocial stressors [21]. Moreover, a 30-year follow-up of females exposed to verified childhood sexual abuse – highly prevalent in EUPD patients [51] – demonstrated associations between altered resting state cortisol levels and EAA [52].

We have recently published findings in a mixed cohort of hypersexual patients and psychiatrically healthy controls (all men), pointing to an independency of EAA to HPA-axis functioning (as measured by an oral low dose overnight dexamethasone test of 0.5 mg) [53]. However, results from other studies paint a somewhat different picture. For example, a study of adolescent girls demonstrated an association between diurnal cortisol and EAA, with EAA mediating an association between diurnal cortisol and reduced hippocampal volume [54]. Importantly, early-life adversity in women is associated with alterations in cortisol regulation that are apparent in adulthood. The independence of self-reports of violence exposure in youth (putative proxy variable to manifest dysfunctional cortisol regulation) to EAA in adulthood observed in the present study, indicate that physiological effects of dysregulated cortisol regulation may not be a substantial contributor to observed EAA increases in severely impaired EUPD patients. Further studies are needed to provide clarity.

Several limitations to this study design prevent causal inferences. First, a longitudinal study-design would have allowed for analyzing the dynamic quality of EA in relation to EUPD. The cross-sectional design implemented for this study does not allow for such analyses. Second, the study lacked in power to comprehensively investigate EAA in relation to EUPD and investigated clinical variables. Thus, it is possible that the demographic variables – for which no association to EAA was reported – confer effects on EAA under the threshold for which the study was able to detect them. However, the present study was sufficiently powered to detect clinically significant EA differences. Assessing EA disparities of small magnitude would require a sizeable cohort of EUPD patients and would, arguably, be unlikely to be relevant to inform clinical practice. Third, a significant and strong inverse association was observed regarding previous or current regular tobacco usage and EAA. Other factors that could be presumed to have an influence on EAA (i.e., substance and/or alcohol abuse, use of psychotrophic medications, age at first suicide attempt) appeared independent of GrimAgeAccel. A plausible explanation lies in the degree of severity of the EUPD group, with relation to suicidal attempts, co-occuring severe mental disorders and high frequency of adverse lifestyle habits (i.e., tobacco usage, history of alcohol or substance abuse). Thus, while the present study due to power limitations is unable to confirm such effects, it cannot be completely excluded – and argued to be more likely than unlikely – that the multitude of putatively interacting negative risk factors on EAA could contribute to mask the impact of individual less-central contributing factors. Lastly, despite controlling for potential confounding factors, residual confounding may exist due to differences between cases and controls on certain covariates. However, it should not influence the association for smoking as this was evaluated in a within-group analysis. Nevertheless, replication of our findings in independent samples would be valuable to confirm and further support our results. Study strengths include the representative patient population of suicide attempters with thorough diagnostics of the psychiatric disorders and a careful assessment of EUPD as well as the consideration of possible confounders such as psychotrophic medications, suicidality, and past substance/alcohol abuse and comorbidity patterns. Moreover, the statistical methods used allow for using peripheral tissues to reliably estimate EA – epigenetic age estimators have been previously demonstrated to be accurately measured from almost any tissue of the body [55].

In conclusion, our results show that epigenetic age acceleration markers previously demonstrated to strongly predict physiological dysregulation and mortality are extensively impacted in severely impaired EUPD subjects. These findings – implicating EA exceeds chronological age by >8 years in female EUPD patients in early adulthood – suggest that the condition and associated adverse lifestyle-habits is associated with excess morbidity and all-cause mortality [56]. Taken together, these findings indicate that a history of EUPD could be considered as a risk-factor for prematurely contracting severe physical illnesses (i.e., CHD, cancer), especially so in subjects with a history of regular tobacco usage. These results underscore the importance of treating somatic health conditions and suggests the use of low-cost preventative interventions to support cessation of tobacco use across the lifespan could be of value in context of EUPD. More generally, the independence of GrimAge to other EA algorithms in this group of severely impaired EUPD patients, suggest it may have unique characteristics to evaluate risk of adverse health outcomes in context of psychiatric disorders.

## Supplementary information

Supplementary figure legends

Supplemental Figure 1

Supplemental Figure 2

## Data Availability

The data and analysis code underlying the findings presented in this study are available upon reasonable request.
