# Supplementary material for: Accelerated epigenetic aging in women with emotionally unstable personality disorder and a history of suicide attempts
Source: Transl Psychiatry. 2023 Feb 22;13:66. doi: 10.1038/s41398-023-02369-7 (PMC9946998; doi:10.1038/s41398-023-02369-7)
Supplement: Supplementary file 1 — Supplementary figure legends [file 41398_2023_2369_MOESM1_ESM.docx]

**Supplementary Figure 1. Difference between baseline *DNAmGrimAge* and chronological age, expressed as percentage of chronological age**

**Figure legend**: Violin plot with boxplots show differences between *DNAmGrimAge* and chronological age, expressed as a percentage of chronological age. The percentage increase in epigenetic age compared to chronological age in EUPD and controls averaged 32.2% and 6.6%, respectively.

**Supplementary Figure 2. Sample size requirements for a power of 0.8 as a function of differences in *DNAmGrimAge***

**Figure legend**: The power.t.test function for R statistics was implemented to evaluate power to detect meaningful differences in AgeAccelGrim between subjects with EUPD and controls. As violin plots were not indicative of any other direction of association, we specified one-tailed hypothesis *t*-tests for the power calculations (evaluating whether EA acceleration measures are greater in the EUPD group). Power-analysis show that the study was sufficiently powered to detect differences of 2.3 years in *AgeAccelGrim* between EUPD subjects and controls for a desired power of 0.8 in one-tailed hypothesis *t*-tests (**Supplementary Figure 2.**), and 2.5 for two-tailed hypothesis *t*-tests (data not illustrated).
