# Supplementary figures and images for: Accelerated epigenetic aging in women with emotionally unstable personality disorder and a history of suicide attempts
Source: Transl Psychiatry. 2023 Feb 22;13:66. doi: 10.1038/s41398-023-02369-7 (PMC9946998; doi:10.1038/s41398-023-02369-7)

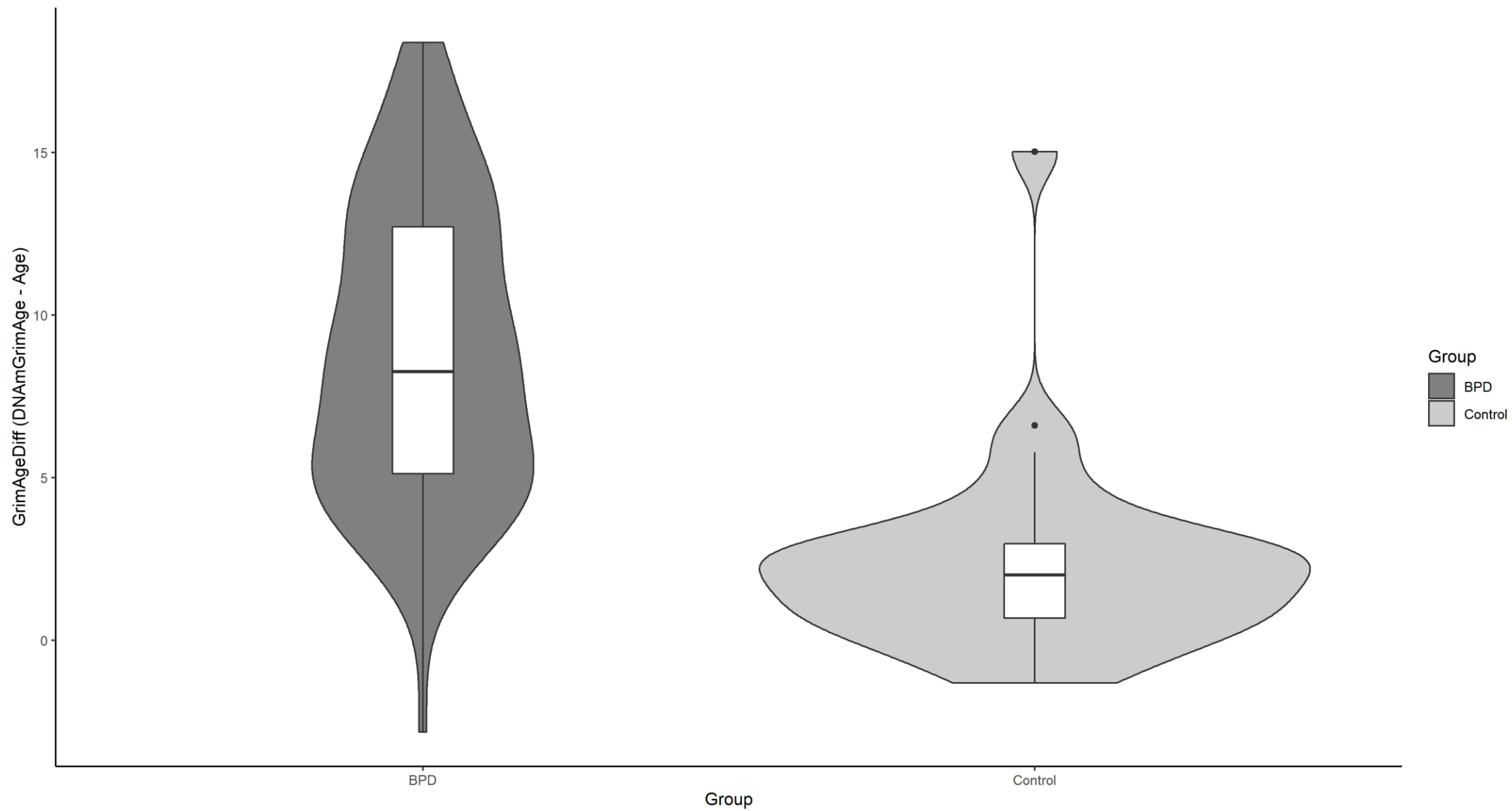

Supplement: Supplementary file 2 — Supplemental Figure 1 [file 41398_2023_2369_MOESM2_ESM.pdf]

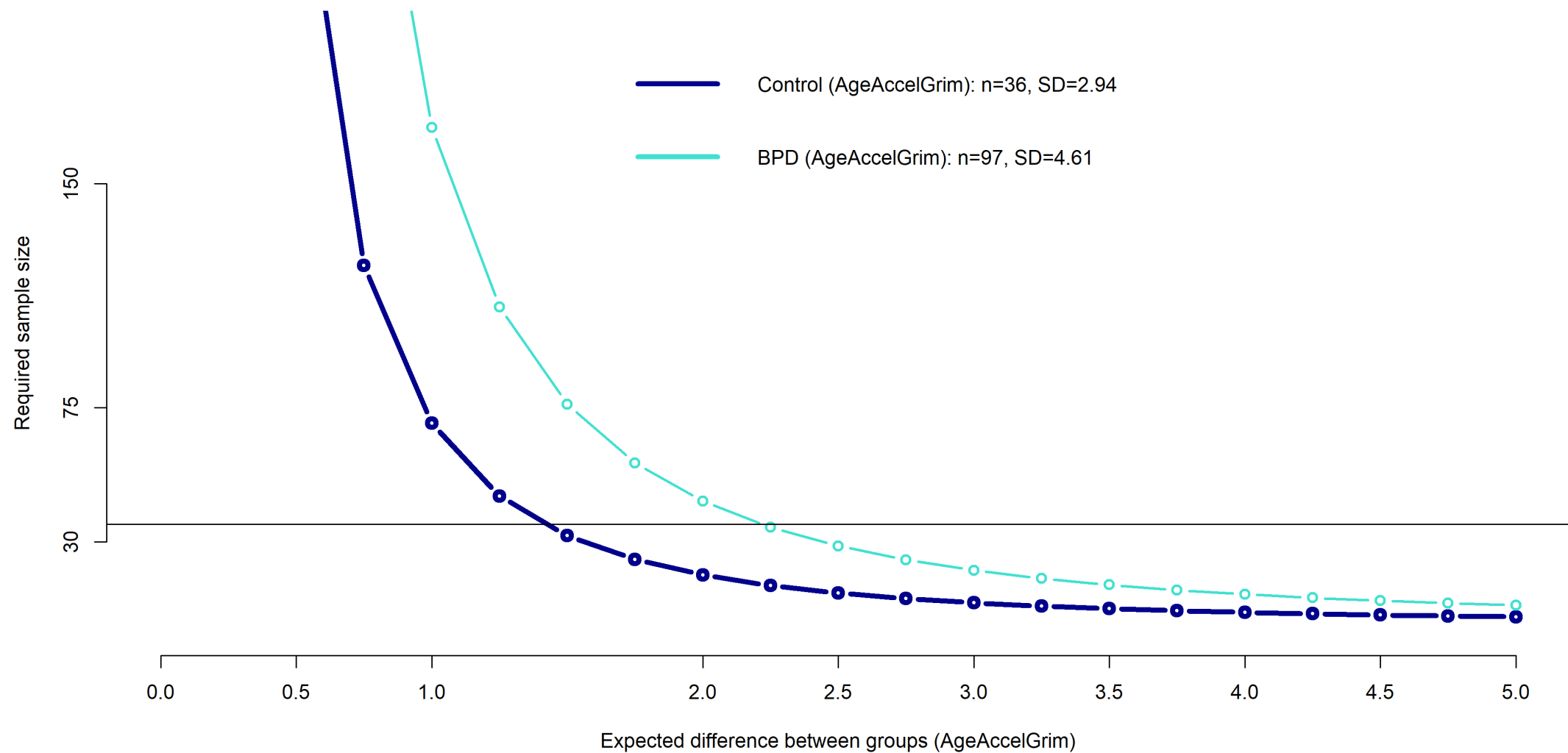

Supplement: Supplementary file 3 — Supplemental Figure 2 [file 41398_2023_2369_MOESM3_ESM.pdf]
